# A Mathematical Model for Predicting Patient Responses to Combined Radiotherapy with CTLA-4 Immune Checkpoint Inhibitors

**DOI:** 10.3390/cells12091305

**Published:** 2023-05-03

**Authors:** Yongjin Kim, Bo-Young Choe, Tae Suk Suh, Wonmo Sung

**Affiliations:** Department of Biomedical Engineering and of Biomedicine & Health Sciences, College of Medicine, The Catholic University of Korea, Seoul 06591, Republic of Korea

**Keywords:** immune checkpoint inhibitor, tremelimumab, radiation therapy, mathematical modeling

## Abstract

The purpose of this study was to develop a cell–cell interaction model that could predict a tumor’s response to radiotherapy (RT) combined with CTLA-4 immune checkpoint inhibition (ICI) in patients with hepatocellular carcinoma (HCC). The previously developed model was extended by adding a new term representing tremelimumab, an inhibitor of CTLA-4. The distribution of the new immune activation term was derived from the results of a clinical trial for tremelimumab monotherapy (NCT01008358). The proposed model successfully reproduced longitudinal tumor diameter changes in HCC patients treated with tremelimumab (complete response = 0%, partial response = 17.6%, stable disease = 58.8%, and progressive disease = 23.6%). For the non-irradiated tumor control group, adding ICI to RT increased the clinical benefit rate from 8% to 32%. The simulation predicts that it is beneficial to start CTLA-4 blockade before RT in terms of treatment sequences. We developed a mathematical model that can predict the response of patients to the combined CTLA-4 blockade with radiation therapy. We anticipate that the developed model will be helpful for designing clinical trials with the ultimate aim of maximizing the efficacy of ICI-RT combination therapy.

## 1. Introduction

Radiation therapy (RT) is traditionally considered a local cancer treatment due to its immune-suppressive characteristics. However, current evidence has demonstrated that radiation also possesses immune-stimulatory characteristics [1,2]. Potential immunogenic cell death via radiation triggered the investigation of radiation combined with immune checkpoint inhibitors (ICIs) for metastatic cancer [3].

A significant concern in adding RT to ICI is the development of radiation-induced immune-suppressive effects [4]. Despite the growing number of trials combining RT and ICI, whether the RT regimen in combination with ICI is the appropriate choice remains an open question, especially considering factors such as the timing of the radiation [5], sequencing [6], and patient selection [7]. Numerous studies [8,9,10,11,12] have investigated the integration of these treatments based on preclinical evidence demonstrating a synergistic interaction between them. However, it remains unclear how to optimally integrate these therapeutic modalities in the treatment of cancer patients. Beyond disease-specific factors, there exist numerous unanswered questions regarding optimal sequencing of radiation and ICI, as well as radiation dosing and target selection [13]. Therefore, it is essential to develop optimal strategies to improve the effectiveness of a combined treatment.

Mathematical models have the potential to help find optimal administration protocols, provide a deeper understanding of the dynamics, and aid in the design of clinical trials [14]. Various mathematical models were developed to simulate the interaction between cancer cells and the immune system. These models aim to predict the outcome of various cancer treatments, such as immunotherapy and radiation therapy. For example, one study investigated the effect of the immunotherapy and radiation therapy dose schedule in terms of immune response time characteristics through a mathematical model and included cyclic cytokine-based immunotherapy treatment in a tumor growth model [15]. Similarly, Sotolongo-Grau et al. proposed a dynamical system model for tumor–immune system interaction together with a method to mimic radiation therapy. A large population of virtual patients was simulated following an ideal radiation treatment. A characteristic parameter, the immune system/tumor efficiency ratio (ISTER), was introduced, and its dependence on treatment success and other features was studied [16]. Other studies have presented a mathematical model for predicting Kaplan–Meier survival curves of combined radiation and chemotherapy in patients with non-small cell lung disease (NSCLC) for use in clinical trial design [17].

In this paper, using a mathematical model, we study the response of a solid tumor to a combined RT and ICI treatment with various treatment regimens. We take hepatocellular carcinoma (HCC) treated with radiation and tremelimumab as a specific example. The first will describe the derivation of a mathematical parameter for CTLA-4 blockade with tremelimumab. The second will describe the patient response to RT and ICI with different treatment regimens, i.e., (1) patient selection and (2) the timing of radiation, in order to achieve maximized tumor eradication for both local and metastatic cancers.

## 2. Materials and Methods

The mathematical model was developed using the Python programming language (Python Software Foundation, Version 3.9, Wilmington, DE, USA).

### 2.1. Model Equations

The previously developed tumor–immune system interaction model was modified to account for the effects of anti-CTLA-4 immune checkpoint inhibitors [18]. The model was applied to predict patient responses after the combined radiation treatment with CTLA-4 immune checkpoint inhibitors.

Our model consists of four compartments—T_I_: irradiated tumor cells, I: dying tumor cells, T_NI_: non-irradiated (metastatic) tumor cells, and L: circulating lymphocytes.

(1) $$\frac{{dT}_{I}}{dt}=aT-\omega_{1}\frac{T_{I}}{g+T_{I}+T_{NI}}L-\delta^{*}\left(t_{R}\right)\left(1-e^{-\alpha_{T}D_{T}-\beta_{T}D_{T}^{2}}\right)T_{I}$$

(2) $$\frac{dI}{dt}{=\delta }^{*}\left(t_{R}\right)\left(1-e^{-\alpha_{T}D_{T}-\beta_{T}D_{T}^{2}}\right)T_{I}-rI$$

(3) $$\frac{dT_{NI}}{dt}=aT_{NI}-\omega_{1}\frac{T_{NI}}{g+T_{I}+T_{NI}}L$$

(4) $$\frac{dL}{dt}{=\omega }_{2}\left(1+\delta_{trem}\right)\frac{T_{I}+T_{NI}}{g+T_{I}+T_{NI}}L+\omega_{3}\frac{I}{g+I}L+s-fL-\delta^{*}\left(t_{R}\right)\left(1-e^{-\alpha_{L}D_{L}}\right)L$$

The new formalism has only one additional term (𝛿_trem_) in Equation (4), describing the effects of CTLA-4 blockade. CTLA-4 blockade demonstrated a mild increase in the circulating lymphocytes, leading to enhanced T cell responses [19]. Thus, the new term (𝛿_trem_) increases the effects of ω_2_, which stimulate the lymphocytes to destroy cancer cells. The effect of the new term (𝛿_trem_) is assumed to decrease exponentially with the decay constant (λ), as in Equation (5). The decay constant is calculated as ln(2) divided by the half-life of tremelimumab.

(5) $$\delta_{trem}=\delta_{0,trem}\cdot e^{-\lambda t}$$

For term ($\delta^{*}\left(t_{R}\right)$), which describes radiation cell death, instead of directly solving the differential equation, radiation cell death is separately accounted for by the instantaneous changes at distinct time points, as shown by the following:

(6) $$T_{n+1}=T_{n}\cdot e^{-\alpha_{T}D_{T}-\beta_{T}D_{T}^{2}}$$

(7) $$I_{n+1}=I_{n}+T_{n}\cdot \left({1-e}^{-\alpha_{T}D_{T}-\beta_{T}D_{T}^{2}}\right)$$

(8) $$L_{n+1}=L_{n+1}{\cdot e}^{-\alpha_{L}D_{L}}$$

Except for the new terms (𝛿_trem_ and λ), other parameters were taken and fixed from the previously developed RT-only model. Table 1 summarizes the resulting average parameters of the population.

The irradiated tumor fraction (ITF) is defined as below:

(9) $$ITF=\frac{T_{I}}{T_{I}+T_{NI}}$$

### 2.2. Patient Cohort

A total of 10,000 virtual patients were implemented into Python programming language for the simulation of a mathematical model. The virtual patient population in this study was assumed to have inoperable HCC. We assumed that each patient had a unique tumor volume, lymphocyte count, and tumor radio-sensitivity. All of the patient population was implemented as a normal distribution using the random syntax method of the Python program. In other words, the patient population had a certain form of distribution in this virtual clinical trial. The same distributions taken from previous studies were implemented in this study. This study assumed the tumor density to be 10^9^ times the number of tumor cells per cubic cm. The averages and standard deviations of each distribution are described in detail in Table 1.

### 2.3. Model Fitting

**Figure 1:**
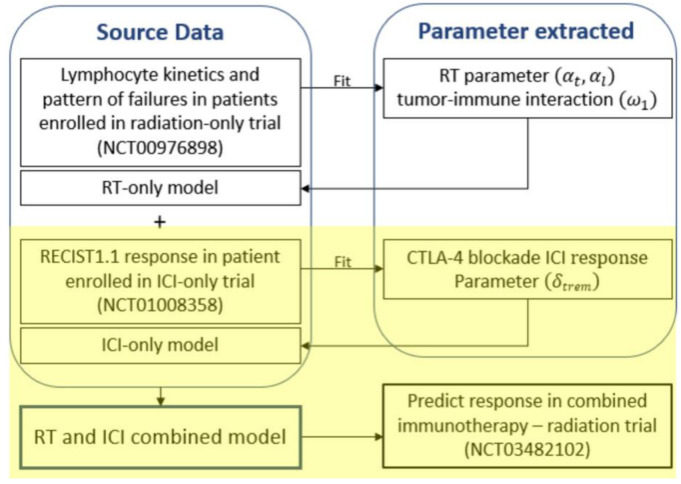
Schematic diagram of model fitting procedure. The yellow highlighted part is a procedure different from the previous study. Abbreviations: RT = radiation therapy; ICI = immune checkpoint inhibitor; RECIST = response evaluation criteria in solid tumors.

Currently, immunotherapeutic agents used clinically show therapeutic effects in only a portion of cancer patients [29]. Therefore, the virtual patient population used in our study adjusted the distribution of the CTLA-4 blockade ICI response using the following parameters. The distribution of an additional new term (𝛿_trem_) in this study is fitted to describe the HCC patient responses to the tremelimumab—a complete response (CR): 0%, partial response (PR): 17.6%, and stable disease (SD): 58.8%. These percentages are based on the response evaluation criteria in solid tumors (RECIST) set of rules 1.1 [28]. To find the distribution, we first calculate the RECIST 1.1 responses for 10,000 patients with a constant 𝛿_trem_ from 0 to 99. In the calculated RECIST 1.1 responses for a constant 𝛿_trem_, we find a distribution suitable for the CTLA-4 blockade ICI response mentioned above using the convolution method. Detailed model fitting procedures are described in Figure 1.

## 3. Results

A mathematical model was developed to predict a patient’s response to the combined radiotherapy with immune checkpoint inhibitors. The model was calibrated based on the responses reported in the CTLA-4 monotherapy clinical trial. We investigated the effects of irradiated tumor burden and treatment sequences under the radiation and CTLA-4 blockade combination regimen.

### 3.1. Calibration

**Figure 2:**
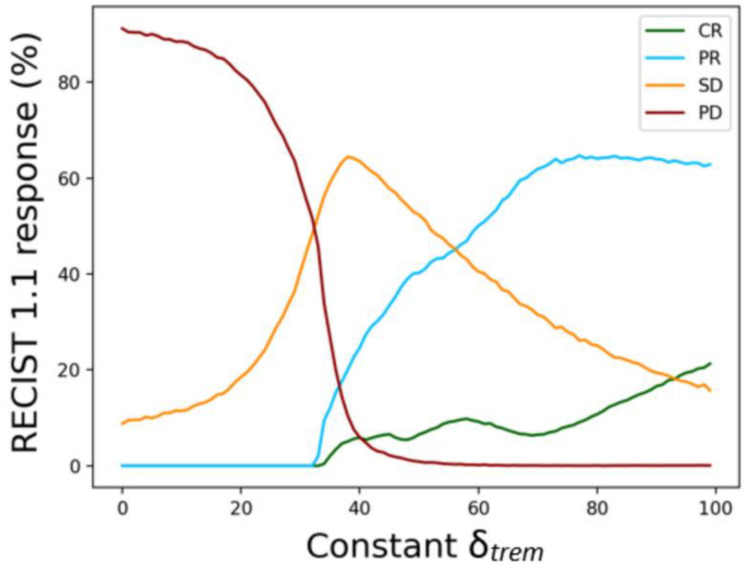
The effect of the 𝛿_trem_ (tremelimumab term) on RECIST1.1 responses for 10,000 virtual patients. The green, blue, orange, and red lines indicate complete response (CR), partial response (PR), stable disease (SD), and progressive disease (PD), respectively. Abbreviations: RECIST = response evaluation criteria in solid tumors.

**Figure 3:**
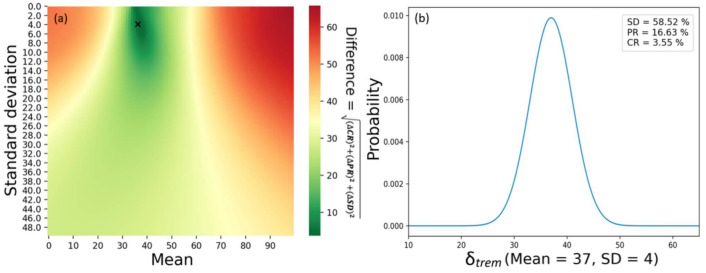
Based on the RECIST 1.1 response to the constant 𝛿_trem_, a figure was used to find the distribution of tremelimumab suitable for the values for PR (17.6%), SD (58.8%), and PD (23.6%) [28]. (**a**) A heatmap showing the difference in relation to the reference according to the mean and standard deviation of tremelimumab. (**b**) Distribution of suitable-for-reference values and RECIST 1.1 responses. Abbreviations: PR = partial response; SD = stable disease; PD = progressive disease; RECIST = response evaluation criteria in solid tumors.

Figure 2 shows the RECIST 1.1 response depending on the fixed 𝛿_trem_ for 10,000 virtual patients. As the 𝛿_trem_ increases, the PD rate decreases, while the PR and CR rates increase at a 𝛿_trem_ value of 35. The SD rate increases up to 65 % at a 𝛿_trem_ value of 40. Based on these results, we are able to set the mean and standard deviation of the 𝛿_trem_ value and achieve RECIST 1.1 responses in patients enrolled in the ICI-only trial (NCT01008358) (Figure 3).

### 3.2. Simulation

**Figure 4:**
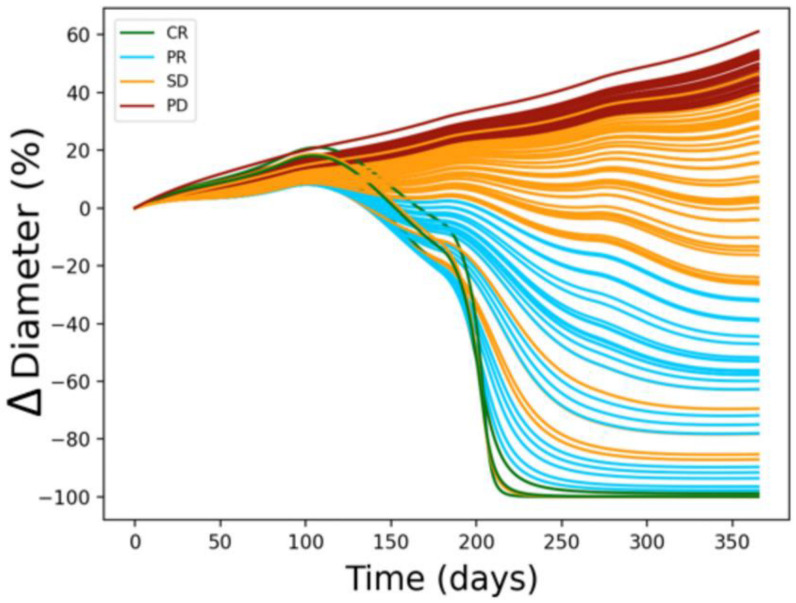
Tumor diameter change over time for 100 virtual patients treated with tremelimumab. Each line represents the tumor dynamics of a single patient. The green, blue, orange, and red lines indicate complete response (CR), partial response (PR), stable disease (SD), and progressive disease (PD), respectively.

Figure 4 shows the relative change in tumor size for 100 virtual patients over time after fitting the 𝛿_trem_ value. One line represents the tumor growth dynamics of a single virtual patient with its corresponding RECIST1.1 response (colors).

**Figure 5:**
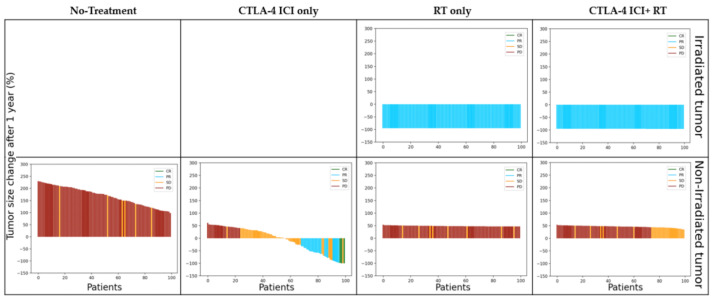
Tumor size changes for 100 virtual patients after one year of treatment (upper: irradiated tumor, lower: non-irradiated tumor). Each bar represents a single patient. The RECIST response 1.1 is expressed as CR: green, PR: blue, SD: orange, and PD: dark red. Abbreviations: CR = complete response; PR = partial response; SD = stable disease; PD = progressive disease; RECIST = response evaluation criteria in solid tumors; RT = radiation therapy; ICI = immune checkpoint inhibitor.

Figure 5 shows the diameter changes in irradiated and non-irradiated tumors for 100 virtual patients. Radiation clearly plays a role in controlling irradiated tumors (waterfall plots on the top in Figure 5). However, radiation led to worse control of the non-irradiated tumors. The CTLA-4-based ICI monotherapy led to the most effective control of non-irradiated tumors. Although adding radiation to ICI achieves better control over irradiated tumors, no complete or partial responses are shown in the non-irradiated tumors.

#### 3.2.1. Irradiated Tumor Burden

**Figure 6:**
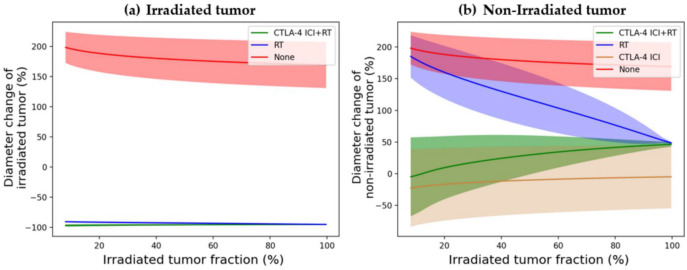
Diameter change in the irradiated tumor and non-irradiated tumor after 1 year according to the irradiated tumor fraction ((**a**): irradiated tumor, (**b**): non-irradiated tumor). The green line indicates CTLA-4 blockade ICI + RT; the blue line indicates RT only; the gold line indicates CTLA-4 blockade ICI only; and the red line indicates none. Abbreviations: RT = radiation therapy; ICI = immune checkpoint inhibitor.

Figure 6 shows results for different treatment regimens in a simulated population of patients with HCC. The results show that the quantitative tumor size changes as a function of the irradiated tumor fraction. The irradiated tumor fraction is applied from 0.1 to 120% to obtain the results. For the irradiated tumor, the irradiated tumor fraction is not a significant factor in reducing the tumor size further. However, for combination regimens, non-irradiated tumor growth increases as the irradiated tumor fraction also increases.

#### 3.2.2. Treatment Sequence

**Figure 7:**
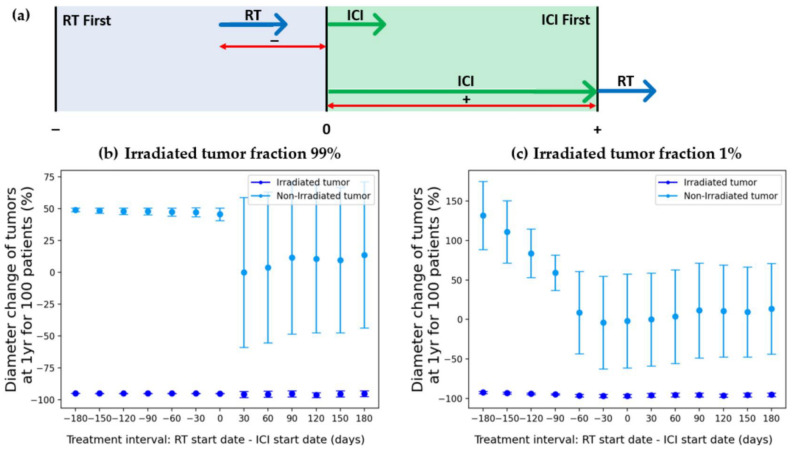
Predicted results of tumor diameter change with different modality combination sequences. (**a**) Schematic to explain (**b**,**c**). Blue and green arrows indicate the start of treatment. On the time axis, negative values (− sign with red arrow in (**a**)) indicate that RT started before the ICI, whereas positive values (+ sign with red arrow in (**a**)) indicate that RT started after the ICI. (**b**) Response to treatment sequence when the irradiated tumor fraction was 99%. (**c**) Response to treatment sequence when the irradiated tumor fraction was 1%. Blue error bars indicate irradiated tumors and light blue error bars indicate non-irradiated tumors. Abbreviations: RT = radiation therapy; ICI = immune checkpoint inhibitor.

We investigated the effects of the sequencing of the RT and ICI combination (Figure 7). The tumor diameter changes were calculated for two simulated patient groups with 99% and 1% irradiated tumor fractions. In both combination cases, the early initiation of CTLA-4 ICI treatment led to the better management of the non-irradiated tumor (plus value of x-axis).

## 4. Discussion

Cancer immunotherapy is now an established part of the therapeutic options for solid tumors. More recently, radiation therapy has shown promising results in augmenting the anti-tumor effects of immune checkpoint inhibition, thus leading to an increased number of clinical trials [30,31,32]. Finding an optimal patient group is crucial not only to improve the treatment responses but also to save limited resources. In principle, the mathematical model is useful for modeling tumor growth under therapy [33]. The actionable model can assist in designing clinical trials by providing thought experiments that investigate potential treatment choices—a process known as an in silico clinical trial. 

### 4.1. Treatment Modality

To confirm which treatment modalities would be effective for tumor control, changes in the size of the irradiated and non-irradiated tumors were obtained for each treatment modality (no treatment, ICI alone, RT alone, RT and ICI combination therapy), confirmed in Figure 5. It can seem that performing CTLA-4 blockade ICI and RT together would generally lead to a better prognosis than performing ICI solely. However, a slightly different result can be confirmed for the non-irradiated tumor size change. When RT is performed alone, the treatment method targets the local area, so it may be thought that there will be little effect on non-irradiated tumors. However, when CTLA-4 blockade ICI and RT are performed together, a worse prognosis can be confirmed than when ICI is performed solely (see the second and fourth graphs at the bottom of Figure 5). This result is thought to be a phenomenon caused by radiation damage to immune cells.

### 4.2. Irradiated Tumor Burden

In Figure 6, the tumor diameter change after 1 year for each treatment modality of the irradiated tumors and non-irradiated tumors was confirmed for the irradiated tumor fraction. The different irradiated tumor burdens lead to different amounts of tumor size changes for each treatment modality. The irradiated tumor burden is not a significant factor in controlling the irradiated tumor. This means that tumor control of the locoregional area is sufficient with RT alone. However, in non-irradiated tumor cases (blue and green lines in Figure 6), when adding CTLA-4-based ICI to RT, the model suggests that the smaller irradiated tumor fraction is better for reducing the non-irradiated tumor.

### 4.3. Treatment Sequence

Figure 7 shows the effect of sequencing and timing on treatment efficacy. The treatment application period of ICI and RT was confirmed at monthly intervals for up to 6 months. As can be seen in Figure 7a, negative numbers indicate that RT was performed first, and positive numbers indicate that ICI was performed first. We found that the responses are maximized if CTLA4-based ICI is delivered earlier than RT. Our results show that opposing behavior is demonstrated with PD-L1-based ICI [14]. This shows that even if it is not the PD-L1 inhibitor, when another inhibitor is used, the sequencing or timing may be different from when the CTLA-4 inhibitor is used. The model shows that early radiation-induced lymphocyte damage is a significant factor when adding CTLA-4-based ICI to RT. Therefore, we applied more indirect anti-tumor effects of CTLA-4-based ICI, reflected in w_3_ in the lymphocyte dynamic equations. Thus, the maintenance of the lymphocyte count is more important in inducing anti-tumor effects in CTLA-4-based ICI, compared to the increased direct effect of PD-L1 (w_1_).

### 4.4. Further Study

In this study, we built a mathematical model and simulated the responses of patients undergoing treatment with CTLA-4 immune checkpoint inhibitors and radiotherapy. There were limitations in our study, such as the weakness of the mathematical model, the inability to simulate various types of cancer, and the inability to simulate other inhibitors. We would like to address the issues in a further study.

## 5. Conclusions

We have developed a mathematical model that can predict patient responses when combined CTLA-4 blockade ICI and RT are performed. Through this model, it is possible to confirm which treatment modality would be effective to apply depending on whether an irradiated tumor or non-irradiated tumor needs to be controlled. It was confirmed that radiation therapy alone was sufficient for irradiated tumor control in a locoregional area, while combination therapy was effective for the metastatic tumor regions without irradiation. In addition, when performing ICI and RT together, it was confirmed that it was better to apply ICI therapy first before RT, and based on the results of Figure 7, we confirmed how much time should be left between treatment modalities in order for the treatment to be more effective. The developed mathematical model can assist in the design of clinical trials through thought experiments that investigate potential treatment choices—a process known as an in silico clinical trial. Therefore, we believe that the developed model can be used for patient selection and designing clinical trials in order to maximize the efficacy of CTLA-4 blockade ICI-RT combination therapy.

## Figures and Tables

**Table 1:** Summary of the parameters. LQ: linear quadratic.

Parameter	Function	Value	Refs.
a	Tumor growth	$0.01\;d^{-1}$	[20]
$\alpha_{T}/\beta_{T}$	Tumor—LQ cell death	14.3 Gy	[21]
f	Lymphocyte decay rate	$0.033\;d^{-1}$	[22]
r	Inactivated tumor cell decay rate	$0.14\;d^{-1}$	[23]
$\omega_{1}$	Tumor-directed lymphocyte efficiency	$0.119\;d^{-1}$	[16,24]
$\omega_{2}$ $\omega_{3}$	Tumor/inactivated tumor lymphocyte recruitment constant	$0.003\;d^{-1}$ $0.009\;d^{-1}$	[16,24]
g	Half-saturation constant	$7.330\times 10^{10}$	[16,24]
s	Lymphocyte regeneration	$1.470\times 10^{8}d^{-1}$	[25]
$\alpha_{T}$	Tumor—LQ cell death	$0.139\;{Gy}^{-1}$	[21]
$\alpha_{L}$	Lymphocytes—LQ cell death	$0.737\;{Gy}^{-1}$	[26,27]
$\delta_{trem}$	Effectiveness of immune checkpoint inhibitor (tremelimumab)	Normally distributed (μ = 37 σ = 4)	[28]

## Data Availability

Data can be obtained from corresponding author upon reasonable request.
